# Deep learning prediction of pathological complete response, residual cancer burden, and progression-free survival in breast cancer patients

**DOI:** 10.1371/journal.pone.0280148

**Published:** 2023-01-06

**Authors:** Hongyi Dammu, Thomas Ren, Tim Q. Duong

**Affiliations:** Department of Radiology, Montefiore Medical Center and Albert Einstein College of Medicine, Bronx, New York, United States of America; Indian Institute of Technology Patna, INDIA

## Abstract

The goal of this study was to employ novel deep-learning convolutional-neural-network (CNN) to predict pathological complete response (PCR), residual cancer burden (RCB), and progression-free survival (PFS) in breast cancer patients treated with neoadjuvant chemotherapy using longitudinal multiparametric MRI, demographics, and molecular subtypes as inputs. In the I-SPY-1 TRIAL, 155 patients with stage 2 or 3 breast cancer with breast tumors underwent neoadjuvant chemotherapy met the inclusion/exclusion criteria. The inputs were dynamic-contrast-enhanced (DCE) MRI, and T2- weighted MRI as three-dimensional whole-images without the tumor segmentation, as well as molecular subtypes and demographics. The outcomes were PCR, RCB, and PFS. Three (“Integrated”, “Stack” and “Concatenation”) CNN were evaluated using receiver-operating characteristics and mean absolute errors. The Integrated approach outperformed the “Stack” or “Concatenation” CNN. Inclusion of both MRI and non-MRI data outperformed either alone. The combined pre- and post-neoadjuvant chemotherapy data outperformed either alone. Using the best model and data combination, PCR prediction yielded an accuracy of 0.81±0.03 and AUC of 0.83±0.03; RCB prediction yielded an accuracy of 0.80±0.02 and Cohen’s κ of 0.73±0.03; PFS prediction yielded a mean absolute error of 24.6±0.7 months (survival ranged from 6.6 to 127.5 months). Deep learning using longitudinal multiparametric MRI, demographics, and molecular subtypes accurately predicts PCR, RCB, and PFS in breast cancer patients. This approach may prove useful for treatment selection, planning, execution, and mid-treatment adjustment.

## Introduction

Neoadjuvant chemotherapy (NAC) [1] is often used to reduce tumor size prior to breast cancer surgery and to minimize distant metastasis with remarkable success. Pathological complete response (PCR) [2, 3] (defined as the absence of any residual disease) and residual cancer burden (RCB) [4] (defined on a scale of 0 to 3 with increasing residual disease burden) are often used to assess NAC response via pathological analysis of biopsied or dissected tissue at the end of the NAC treatment course. Patients with PCR or low RCB scores are more likely to be candidates for breast-conserving surgery sparing a full mastectomy and are also likely to have longer progression-free survival (PFS) and overall survival [2, 3]. The ability to longitudinally monitor individual response to NAC and to determine patient’s likelihood to respond to NAC early on in the treatment course is clinically important because it could help to minimize unnecessary toxic NAC and modify regimens mid-treatment to achieve better efficacy. A major challenge to date is the lack of reliable methods to assess efficacy early in the NAC course.

Breast MRI is a standard of care for cancer diagnosis, staging, prognosis and treatment monitoring MRI can non-invasively identify in-breast cancer with excellent accuracy and specificity [5, 6]. Many studies have reported using radiological staging, MRI tumor volume, and radiomic features from pretreatment MRI to predict PCR, RCB and PFS [7, 8]. Although promising, identifying reliable imaging and non-imaging metrics to predict PCR, RCB and PFS remains an active area of research.

Machine-learning has become increasingly popular for image classification and prediction [9–11]. One common deep-learning algorithm is the convolutional neural network (CNN), which takes an input image, learns important features in the image such as size, shape, or intensity, and saves these model’s parameters as weights and bias to differentiate different types of images [12]. While many studies have applied machine-learning on extracted radiomic features (such as volume, sphericity, dynamic contrast enhanced (DCE) MRI signal of wash in and wash out) as inputs to predict PCR [13–16] [reference] (see review paper [7, 8]), only a few have applied deep learning to predict PCR using whole MR images [17–19], DCE dynamics [20], inclusive of non-imaging clinical data such as demographics and molecular receptor subtypes [21, 22], and/or multiple time points during treatment (see review (refernce)). Even fewer reported deep-learning prediction models to predict RCB and PFS [7]. To our knowledge, there have been no studies using deep learning that combine whole breast MRI, DCE MRI dynamics, MRI at multiple treatment time points, and inclusion of non-imaging data to predict RCB and PFS.

The goal of this study was to develop a novel deep-learning CNN method to predict PCR, RCB, and PFS in breast cancer patients treated with neoadjuvant chemotherapy. This novel (referred to as Integrated CNN) deep learning method takes a whole-breast DCE MRI at multiple time points during neoadjuvant chemotherapy as inputs. Moreover, non-imaging data, such as demographics and molecular subtypes, are also fed into the models. Performance was evaluated using receiver-operating characteristic analysis. For comparison, results were compared with two more conventional methods, referred to as “stacking” and “concatenation” CNN methods.

## Materials and methods

Institutional review board approval is not required. Codes are available via https://github.com/HongyiDuanmu26/Prediction-of-pCR-with-Integrative-Deep-Learning. I-SPY-1 data used in this paper are available via the https://www.cancerimagingarchive.net

### Data sources

Level 3 curated data from the I-SPY-1 TRIAL (2002–2006) were used in this analysis [23, 24]. All patients were diagnosed with stage 2 or 3 breast cancer with breast tumors at least 3 cm in size and underwent anthracycline-cyclophosphamide (AC) with or without Taxane treatment. The I-SPY TRIAL 1 Level 3 dataset included 221 patients with non-imaging variables. We further excluded 66 patients due to missing MR images for all four time points. The final sample size was N = 155 from nine different institutions.

Imaging data included dynamic contrast enhanced (DCE) MRI data obtained at the four time points: a) within four weeks prior to starting anthracycline-cyclophosphamide chemotherapy (time point 1, TP1), b) at least 2 weeks after the first cycle of AC and prior to the second cycle of AC (TP2), c) between anthracycline-cyclophosphamide treatment and Taxane therapy if Taxane was administered (TP3), and d) after the final chemotherapy treatment and prior to surgery (TP4). Each DCE MRI data had three dynamics.

Non-imaging data included demographic data (age, race), estrogen receptor status (ER), progesterone receptor status (PR), human epidermal growth factor receptor 2 (HER2) status, 3-level hormonal receptor (HR)/HER2 category, and Ki-67 (**Table 1**).

**Table 1:** Demographics and clinical features used in the prediction models.

	Description	Statistics
Age	Patient Age (Years)	Mean 48.4±8.9
Race	1 = Caucasian	1 = 118 (75.13%)
	2 = African American	2 = 0 (0.00%)
	3 = Asian	3 = 27 (17.42%)
	4 = Native Hawaiian/Pacific Islander	4 = 8 (5.16%)
	5 = American Indian/Alaskan Native	5 = 1 (0.65%)
	6 = Multiple race	6 = 1 (0.65%)
Estrogen Receptor (ER)	0 = Negative	0 = 69 (44.52%)
	1 = Positive	1 = 84 (54.19%)
	-1 = Indeterminate	-1 = 2 (1.29%)
Progesterone Receptor (PR)	0 = Negative	0 = 88 (56.77%)
	1 = Positive	1 = 65 (41.94%)
	-1 = Indeterminate	-1 = 2 (1.29%)
HER2 Status	0 = Negative	0 = 67 (43.23%)
	1 = Positive	1 = 86 (55.48%)
	-1 = indeterminate or not done	-1 = 2 (1.29%)
3-level HR/HER2 category	1 = HR Positive, HER2 Negative	1 = 63 (40.65%)
	2 = HER2 Positive	2 = 50 (32.26%)
	3 = Triple Negative	3 = 38 (24.52%)
	-1 = indeterminate or not done	-1 = 4 (2.58%)
Ki-67	1 = <10%	1 = 38 (20.65%)
	2 = 10–20%	2 = 37 (23.87%)
	3 = >20%	3 = 57 (36.77%)
	-1 = indeterminate or not done	-1 = 23 (14.84%)

Outcome variables included PCR, RCB, and PFS: i) PCR is either 0 or 1 where 1 indicates pathological complete response, ii) RCB includes 0, 1, 2, and 3 where 0 indicates no residual disease burden and 1–3 indicates increasing residual disease burden, and iii) PFS is a continuous variable measured in months. The sample sizes for different outcomes are shown in **Table 2**.

**Table 2:** Sample sizes for different clinical outcomes based on input and prediction score.

Ground truths	Inputs	Score	N
PCR	MRI + Non-MRI	0	110
		1	42
RCB	MRI + Non-MRI	0	41
		1	14
		2	61
		3	30
PFS	MRI + Non-MRI	months	155

### System architecture

**Fig 1:**
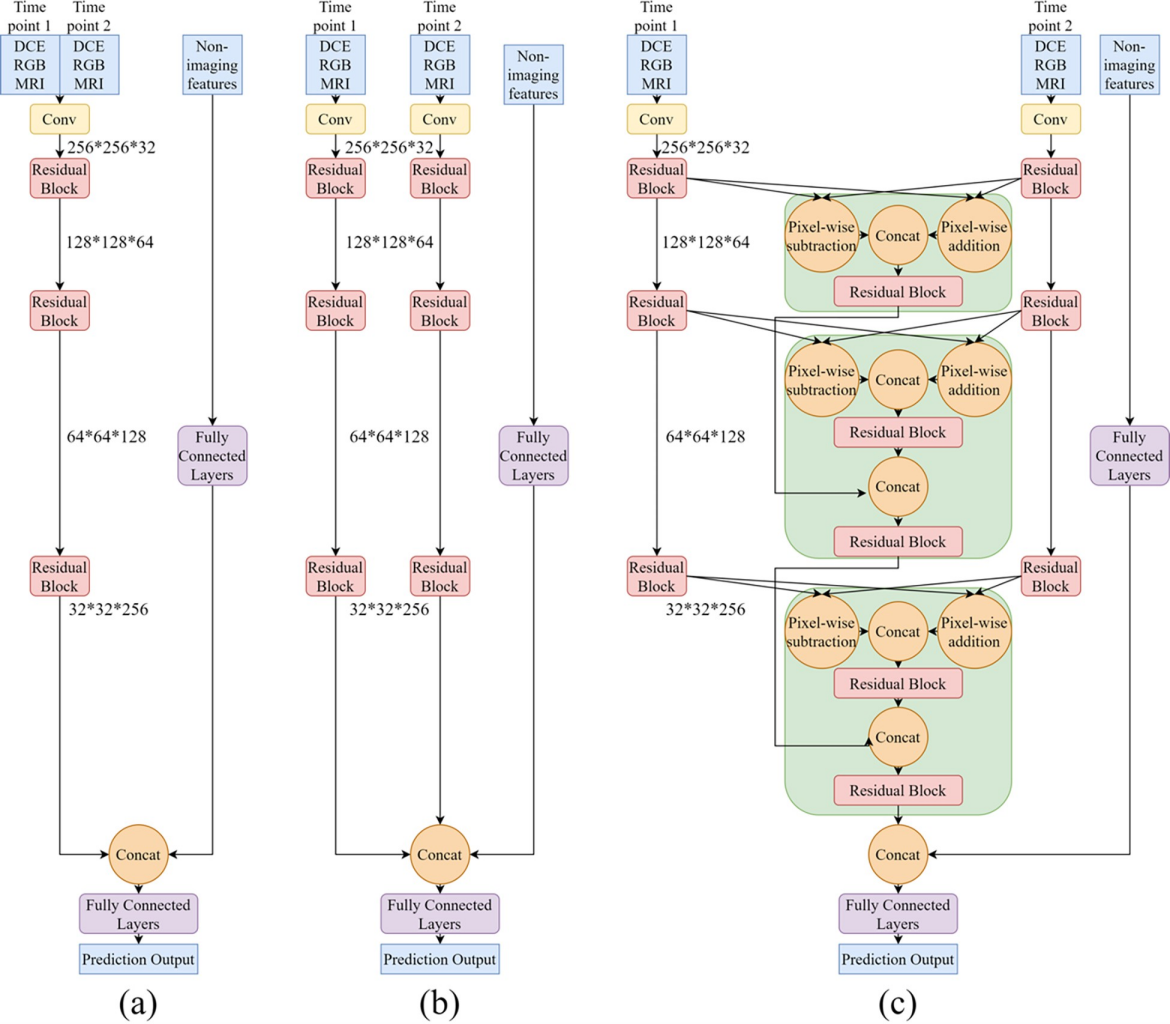
CNN architectures. a) Stack, b) Concatenate, and c) our novel Integrated approach.

The composite RGB images were derived from the three dynamic DCE MRI images and displayed as red, green, and blue. The DCE 3D MRI (not multi-slice) without tumor segmentations were fed into the CNN. As shown in **Fig 1**, DCE image inputs were first fed into a convolutional layer with stride 2 to reduce the dimension of the image array (yellow block). Then, coarse features were processed by three residual blocks sequentially for capturing refined image features and patterns (red blocks) [25]. Each residual block included four convolutional layers, three as the main branch and one as a shortcut identity connection. Residual block has been proven to be beneficial to the robustness of the system and the stability of training [25]. The output from the main branch pixel-wise added with the output from the identity connection was set as the output of the residual block. Non-imaging features were first processed by three fully connected layers, then concatenated with the image features extracted from MR images. Two fully connected layers were used to process the concatenated imaging and non-imaging features for the final clinical outcome prediction. Throughout the whole system, one batch normalization layer and one parametric rectified linear unit (PReLU) activation layer were deployed after each convolutional and fully connected layer.

**Fig 1A** shows the CNN system architecture using the “Stack” method to integrate multiple MR images into the outcome prediction. “Stack” works simply by overlaying two MR images at different time points. The stacked MR images are then fed into one ResNet-based CNN prediction system illustrated above. **Fig 1B** shows the architecture of the system using “Concatenation” method to combine MR images at two different time points. “Concatenation” method joins a sequence of tensors in high-level which is processed by several convolutional layers while ‘Stack’ method combines them before feature extraction. In both methods, MR images at two time points are fed into two symmetric branches and combined using separate approaches. These two methods are easy to implement but not able to fully explore the information between two time points, setting as the baseline model for comparison.

**Fig 1C** shows the “Integrated” approach which we proposed to fully utilize the temporal information. Similarly, ‘Integrated’ method takes MRI images from two treatment time points into two convolutional branches. Then the feature maps extracted from images in the Residual block were fed into one newly proposed block. In each *block* (green), pixel-wise addition and pixel-wise subtraction were calculated for the two inputs from different time points, representing the average and difference information of two inputs from two time points, respectively. After pixel-wise processing, two outputs were concatenated and fed into a residual block to refine the features. The outputs from this *block* were concatenated with the outputs from the previous *block*, integrating longitudinal information extracted from the last level of features. Finally, these output values were processed through another residual block before being pushed to the next block for further processing. The *block* feeds the two images through two residual blocks and concatenates the images from the previous *block* to emphasize important temporal features. In contrast, the “Stacking” and the “Concatenation” simply combine images across a particular dimension, but these specific temporal features are not utilized. To predict PCR, RCB, and PFS, the output dimensions of the last fully connected layer were changed accordingly.

RGB MRI images and non-imaging data were used as inputs unless otherwise noted. For PCR prediction, seven different MRI inputs were used: a) TP1, b) TP2, c) TP3, d) TP4, e) TP1+TP2, f) TP1+TP3, and g) TP1+TP4. In the first four conditions, the model only processed a single time point MRI. For the multiple MRI time point inputs, three different prediction methods: stack, concatenate and integrated approaches were applied. In addition, we also evaluated the integrated approach without using non-imaging data. For the RCB and PFS prediction, only the integrated approach was used.

### Performance evaluation

Five-fold cross-validation was used. One-fifth of the data was held out for validation, and the remaining four-fifths were used to train the models. This was cycled and repeated four additional times to generate 5 independent datasets splits. The model weights saved from the training set were used to predict outcomes on the validation dataset. Batch sizes of 8 were used to limit computational expense and the system was trained for 100 epochs. Several optimizers were tested; however, stochastic gradient descent with momentum gave the lowest validation loss. Nesterov momentum was enabled for more stable training. The learning rate was set to 0.001 and the momentum was set to 0.9. Categorical cross entropy was used as the loss function in PCR and RCB prediction as these two tasks were classification tasks while mean square error was used as the loss function in PFS prediction as it was a regression task. All experiments shared the same training hyperparameter configurations. The evaluation of prediction performance used standard ROC analysis of the area under the curve (AUC), accuracy, sensitivity, specificity, F1 score, Cohen’s kappa coefficient, and mean absolute error (MAE) to provide a general measure of the model performance.

### Statistical analysis

In terms of Statistical comparison, the T-test was used to evaluate if the means of the performance between the two methods are statistically different from each other. Under the T-test, if the difference between the two methods is different with a high degree of confidence (95%), we conclude that the performance of one model is statistically better than that of the other one.

## Results

**Fig 2:**
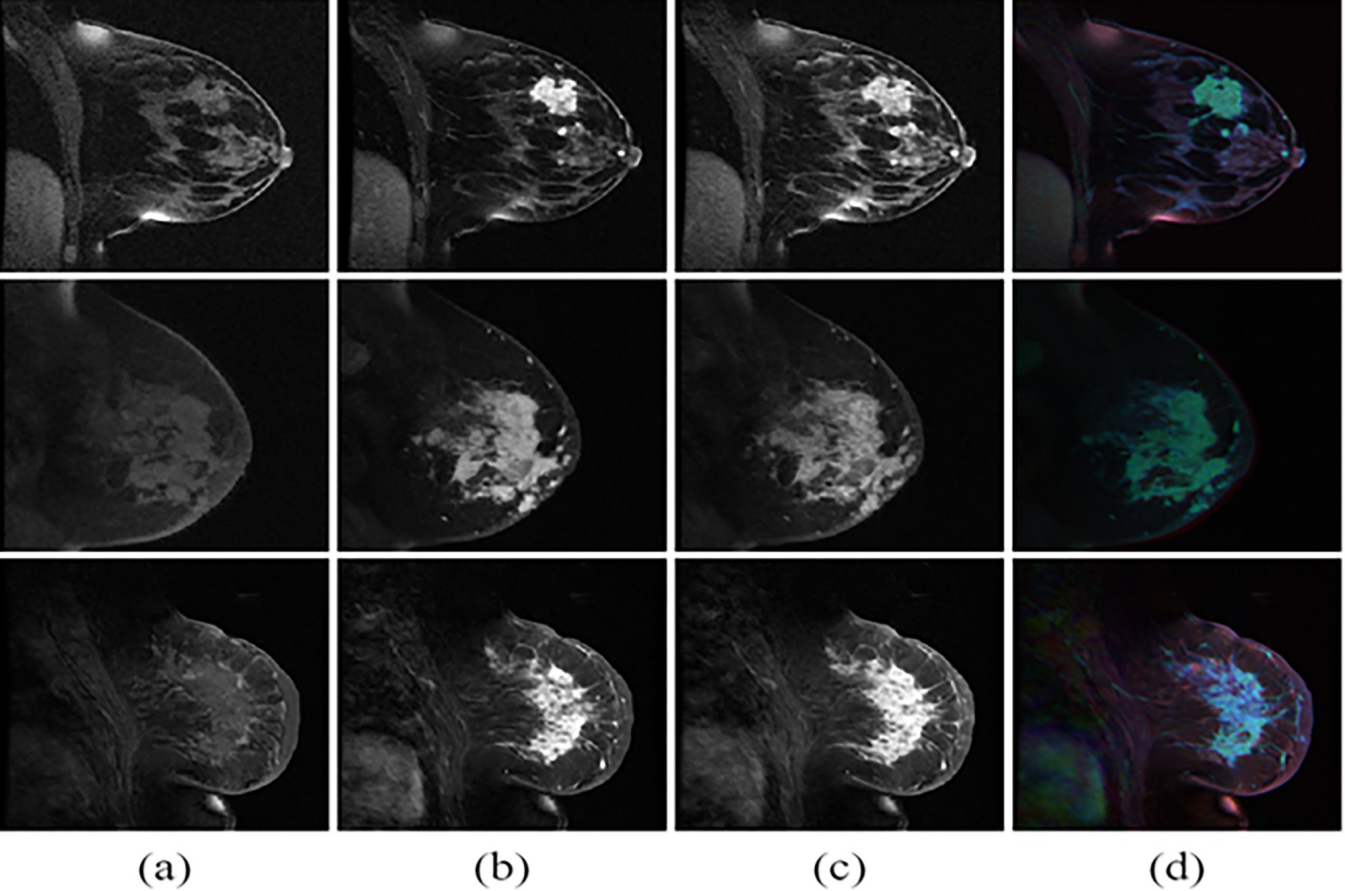
Three phase DCE MR images from three typical patients. (a) phase I, b) phase II, and c) phase III, and (d) the composite image from DCE dynamics displayed as color. Regions in which the first dynamic are brighter appear redder, regions in which the second dynamic are brighter appear greener, and regions in which the third dynamic are brighter appear bluer.

**Fig 2** shows the three dynamic DCE MRIs and the derived composite images in RGB color for three patients. Depending on tissue types, signal intensity changes differently across the three dynamics. Specifically, tumor tissue usually increased signal intensity rapidly and to a greater magnitude. For the DCE images, regions where the first dynamic of the DCE MR images are brighter appear red. Regions where the second dynamic of the DCE MR images are brighter appear green. Regions where the third dynamic of the DCE MR images are brighter appear blue. The composite image fed into the CNN model, making it possible for systems to extract patterns among three dynamics.

### PCR prediction

We evaluated the performances of three methods to predict PCR using DCE MRI images at both TP1 and TP4 treatment time points, as well as non-imaging data as inputs (**Table 3**). The “Integrated” approach performed markedly better in accuracy, AUC and F1, followed by the “Concatenation” and the “Stack” method. The best model yielded an accuracy of 0.81±0.03 and an AUC of 0.83±0.03. The “Integrated” model outperformed the Stack model (p<0.05, t-test) and Concatenation model (p<0.05). To evaluate the relative contribution of image and non-image data, the Integrated approach using image data only without the non-imaging data was also evaluated. The performance indices (accuracy of 0.71±0.08 and AUC of 0.67±0.08) were worse than those with both imaging and non-imaging data.

**Table 3:** Prediction performance comparison between using stack, concatenation, and novel difference block.

	Accuracy	AUC	Sensitivity	Specificity	F1 score
**Stack approach**	0.70 (0.06)	0.61 (0.13)	0.26 (0.17)	0.86 (0.08)	0.45 (0.10)
**Concatenation approach**	0.75 (0.09)	0.60 (0.10)	0.16 (0.21)	0.97 (0.04)	0.37 (0.12)
**Integrated approach**	0.81 (0.03) *	0.83 (0.03) *	0.68 (0.08) *	0.86 (0.04)	0.76 (0.04)*
**Excluding non-imaging data** ^**$**^	0.71 (0.08)	0.67 (0.08)	0.43 (0.04)	0.88 (0.04)	0.56 (0.05)

An “Integrated” approach was also performed excluding non-imaging data. Means and standard deviations (in parentheses) were calculated across five-fold cross validations.

* indicates statistical significance between TP1+TP4 versus all the others (pair wise).

^$^ indicates analysis was performed excluding non-imaging data for the Integrated approach only.

The performances using different time points data with images and non-imaging data were also evaluated (**Table 4**). For the single time point data, TP4 performed overall better than TP1, TP2, and TP3 alone, as defined by accuracy and AUC. For two time point data, TP1+TP4 performed better than TP1+TP2 (p<0.05) and TP1+TP3 (p<0.05), defined by accuracy and AUC. Note that the best model (TP1+TP4) was the same as the best model in **Table 3**.

**Table 4:** PCR prediction performance comparison.

	Accuracy	AUC	Sensitivity	Specificity
TP1	0.76 (0.01)	0.70 (0.02)	0.45 (0.18)	0.86 (0.07)
TP2	0.74 (0.01)	0.69 (0.03)	0.38 (0.22)	0.87 (0.10)
TP3	0.75 (0.01)	0.69 (0.03)	0.33 (0.19)	0.91 (0.07)
TP4	0.77 (0.03)	0.78 (0.04)	0.49 (0.06)	0.88 (0.04)
TP1+TP2	0.76 (0.02)	0.73 (0.04)	0.46 (0.13)	0.88 (0.05)
TP1+TP3	0.76 (0.03)	0.76 (0.03)	0.39 (0.18)	0.90 (0.03)
TP1+TP4	0.81 (0.03)*	0.83 (0.03)*	0.68 (0.08)*	0.86 (0.04)

Mean and standard deviation in parentheses were calculated among five-fold cross validations.

* indicates statistical significance between TP1+TP4 versus all the others (pair wise).

### RCB prediction

The performances of the “Integrated” approach to predict RCB using multiple time point data were also evaluated (**Table 5**). For the single time point data, TP4 performed overall better than TP1, TP2, and TP3 alone. TP1+TP4 performed better than TP1+TP2 (p<0.05), and TP1+TP3 (p<0.05). The best RCB prediction was the TP1+TP4 model yielding an accuracy of 0.80±0.02 (Cohen’s κ = 0.73±0.03).

**Table 5:** RCB prediction performance comparison.

	Accuracy	Cohen’s κ
TP1	0.71 (0.02)	0.59 (0.03)
TP2	0.65 (0.04)	0.51 (0.04)
TP3	0.68 (0.02)	0.56 (0.03)
TP4	0.76 (0.03)	0.68 (0.04)
TP1+TP2	0.69 (0.04)	0.57 (0.07)
TP1+TP3	0.71 (0.03)	0.60 (0.04)
TP1+TP4	0.80 (0.02)*	0.73 (0.03)*

Mean and standard deviation in parentheses were calculated among five-fold cross validations.

* indicates statistical significance between TP1+TP4 versus all the others (pair wise).

### PFS prediction

**Fig 3:**
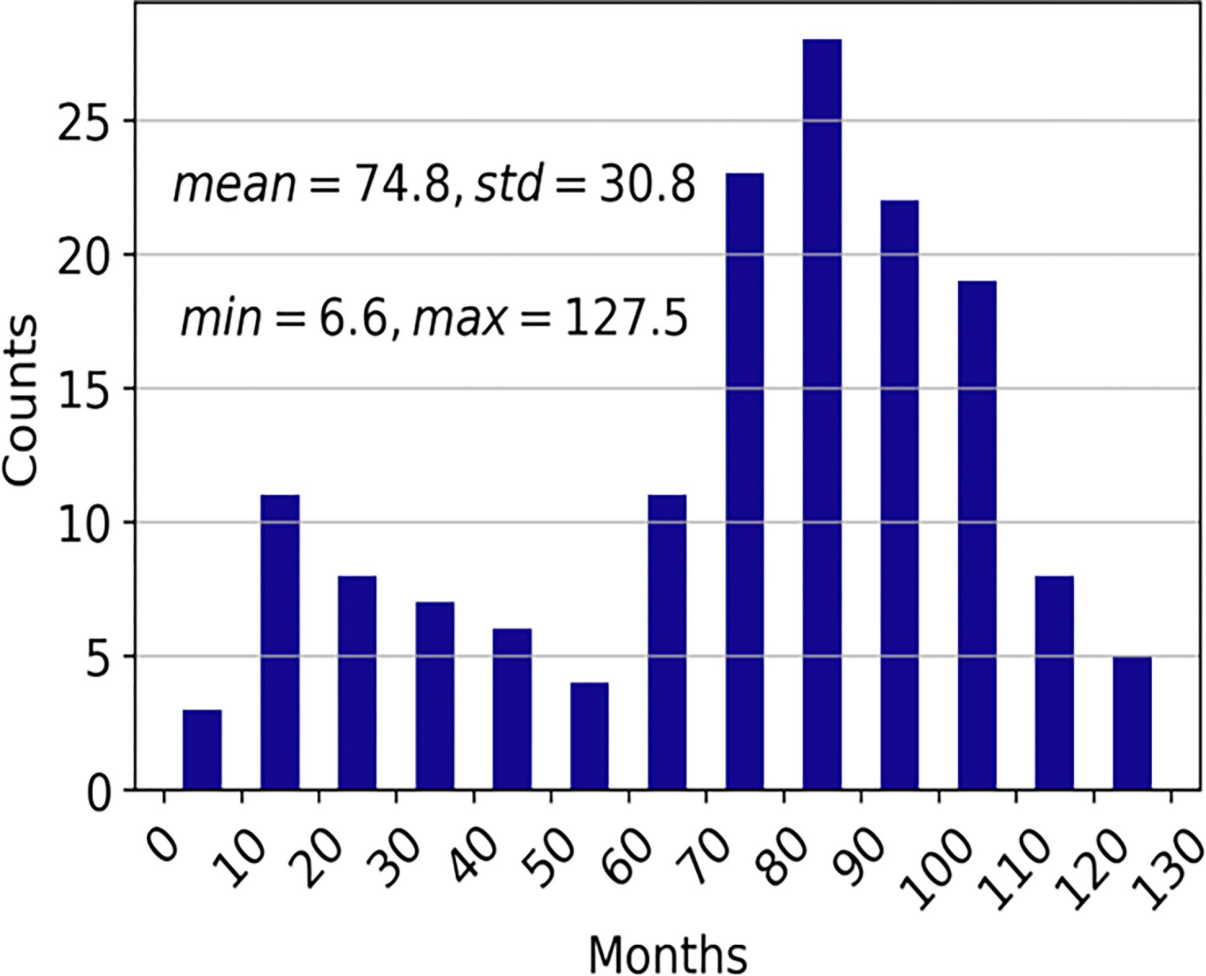
Histogram of progression free survival (PFS) in months (N = 155).

The PFS in months was 74.8±30.8 months (range: 6.6 to 127.5) (**Fig 3**). The MAEs of PFS prediction are summarized in **Table 6**. For the single time point data, TP4 performed overall better than TP1, TP2, and TP3 alone (p<0.05). TP1+TP4 performed better than TP1+TP2 (p<0.05) and TP1+TP3 (p<0.05). The best model (TP1+TP4) predicted PFS within 24.6±0.7 months (MAE).

**Table 6:** Prediction performance of progression free survival as mean absolute error (MAE).

	MAE
TP1	41.6 (2.0)
TP2	48.8 (3.9)
TP3	47.6 (1.5)
TP4	35.9 (2.6)
TP1+TP2	45.1 (7.5)
TP1+TP3	41.5 (5.1)
TP1+TP4	24.6 (0.7)*

Mean and standard deviation (in parentheses) are calculated among five-fold cross validations.

* indicates statistical significance between TP1+TP4 versus all the others (pair wise).

## Discussion

We developed and evaluated three CNN approaches that combined multiple treatment time points of 3D whole-breast DCE MRIs and non-MRI clinical data as inputs to predict PCR, RCB, and PFS in breast cancer patients treated with neoadjuvant chemotherapy. The major findings are: i) the Integrated approach to combine multiple treatment time points of whole-breast DCE MR data outperforms the “Stack” or “Concatenation” approach, ii) inclusion of both MRI and non-MRI data outperformed either alone, iii) prediction using the combined pre- and post-NAC data generally yields better performance compared to prediction using either alone, iv) using the best models, PCR prediction yielded an accuracy of 0.81±0.03 and AUC of 0.83±0.03; RCB prediction yielded an accuracy of 0.80±0.02 and Cohen’s κ of 0.73±0.03; and PFS prediction yielded an MAE of 24.6±0.7 months (patients survival ranged from 6.6 to 127.5 months).

### PCR

Although many studies have reported machine learning methods to predict PCR (see reviews [7, 8]), only a few studies used deep learning on whole MRI images as inputs to predict PCR [17, 26–30]. **Liu et al.** used a 12-layer CNN to analyze patients from the I-SPY trial dataset to predict PCR in NAC patients (N = 131) [28] using 2D MR images with three DCE phases. They reported an accuracy, sensitivity, specificity, and ROC AUC of 72.5%, 65.5%, 78.9%, and 0.72 respectively. **Huynh et al.** utilized CNN and Linear Discriminant Analysis (LDA) classifier to predict response to neoadjuvant chemotherapy in breast cancer patients (N = 64) [29] using 2D DCE MR images. Features which were first extracted using CNN were then used to train the LDA classifier. They found the best ROC AUC was 0.85 for the pre-contrast DCE. A limitation of this study is that slices containing the tumor were manually selected. **El Adoui et al.** applied a 3D CNN to predict PCR from DCE-MRI (N = 42) [30] using multiple treatment time points. Using a two-branch CNN model to take inputs from MRI pre- and post-chemotherapy, they found an accuracy and ROC AUC of 91.03% and 0.92, respectively. **Qu et al.** applied a 2D CNN to predict PCR from multiple DCE MR images plus molecular subtypes including ER, PR, and HER2 (N = 302) [27]. This model used 12 channels to combine 6 DCE phases from both pre- and post-NAC. They reported an AUC of 0.553. A limitation is that pre-NAC and post-NAC MR images were included by concatenation and thus the temporal information might not be optimally utilized. Our study is novel because it utilized deep learning to predict PCR operating on three-dimensional images on the entire breast images without the need to manually segment tumor. Moreover, multiple treatment time points and multi-phase dynamic contrast images were used to improve performance. We also used demographics and molecular subtypes in the model to improve prediction performance. Finally, our CNN model integrating these inputs is also innovative.

### RCB

RCB provides a dynamic range of disease burden in contrast to the binary outcome of PCR [31]. There are a few studies that have used logistic regression and supervised machine learning methods to predict RCB [23, 32]. **Hylton et al.** utilized univariate and multivariate logistic regression models to predict RCB in breast cancer patients’ post-NAC [23] (N = 216) using MRI, pathology reports and slides, and non-imaging data. They explored multiple time point analyses in their models with the best AUC of 0.80. **Tahmassebi et al.** evaluated a few supervised machine learning methods (including support vector machine (SVM), linear regression, and random forest) to predict RCB (N = 38) and found that the best performance was achieved by XGBoost with an AUC of 0.86 [32]. However, there have been no deep learning studies that combine whole breast image, DCE dynamics, multiple treatment time points, and inclusion of non-imaging data to predict RCB to our knowledge. Our study is novel. Our best RCB prediction using multiple treatment time points along with 3D MRI, and DCE and non-imaging data yielded an accuracy of 0.80±0.02 (Cohen’s κ = 0.73±0.03).

### PFS

Although many studies have reported the use of clinical features, pathology images, or radiomic features to predict PFS, few used machine learning of MRI to predict PFS (see review [33]). **Tahmassebi et al.** explored supervised machine learning methods to predict PFS (N = 38) with the best performance achieved by linear regression with an AUC of 0.83 [32]. **Shouket et al.** applied supervised machine learning to predict PFS (N = 200) with an AUC of 0.881, [34] in which PFS was dichotomized in > or < 5 years. Our study predicted PFS in months and reported metric in mean absolute errors using deep learning. Our best model predicted PFS within 24.6±0.7 months mean absolute errors (PFS ranged from 6.6 to 127.5 months).

### Limitations

To our knowledge, this is the deep learning approach to combine whole breast MRI, DCE MRI dynamics, MRI at multiple treatment time points, and non-imaging data to predict PCR, RCB, and PFS. Our study has several limitations. This study was performed on a relatively small dataset and these findings need to be replicated on a larger dataset to improve the generalizability. Other studies have incorporated axillary lymph node MRI in predicting PCR [35–37]. Future studies could also incorporate axillary lymph node MRI and other data to boost the performance.

## Conclusions

We implemented an innovative deep learning CNN model that combines whole breast MRI, DCE MRI dynamics, T2-weighted MRI, MRI at multiple treatment time points, and inclusion of non-imaging data to predict RCB and PFS in breast cancer patients treated with neoadjuvant chemotherapy. This approach can be used to identify patients who are likely to respond to neoadjuvant chemotherapy at diagnosis or early treatment and may prove useful for treatment planning, treatment execution, and mid-treatment adjustment.

## List of abbreviations

CNN: Convolutional neural network
PCR: Pathological complete response
NAC: Neoadjuvant chemotherapy
RCB: Residual cancer burden
PFS: Progression-free survival
AI: Artificial intelligence
ROC AUC: Receiver operating characteristic area under curve
^18^FDG: ^18^Fluorodeoxyglucose
DCE MRI: dynamic contrast enhanced MRI
TP1: time point 1
ER: Estrogen receptor status
PR: Progesterone receptor status
HER2: Human epidermal growth factor receptor 2
PReLUs: parametric rectified linear units
AUC: Area under the curve
MAE: mean absolute error
SVM: support vector machine
